# Targeting Tie2 in the Tumor Microenvironment: From Angiogenesis to Dissemination

**DOI:** 10.3390/cancers13225730

**Published:** 2021-11-16

**Authors:** Camille L. Duran, Lucia Borriello, George S. Karagiannis, David Entenberg, Maja H. Oktay, John S. Condeelis

**Affiliations:** 1 Department of Anatomy and Structural Biology, Einstein College of Medicine/Montefiore Medical Center, Bronx, NY 10461, USA; camille.duran@einsteinmed.org (C.L.D.); lucia.borriello@einsteinmed.org (L.B.); david.entenberg@einsteinmed.org (D.E.); moktay@montefiore.org (M.H.O.); 2 Gruss-Lipper Biophotonics Center, Einstein College of Medicine/Montefiore Medical Center, Bronx, NY 10461, USA; georgios.karagiannis@einsteinmed.org; 3 Department of Microbiology and Immunology, Einstein College of Medicine/Montefiore Medical Center, Bronx, NY 10461, USA; 4 Integrated Imaging Program, Einstein College of Medicine/Montefiore Medical Center, Bronx, NY 10461, USA; 5 Department of Pathology, Einstein College of Medicine/Montefiore Medical Center, Bronx, NY 10461, USA; 6 Department of Surgery, Einstein College of Medicine/Montefiore Medical Center, Bronx, NY 10461, USA

**Keywords:** Tie2, angiopoietin, tumor microenvironment, metastasis, angiogenesis, dissemination, TMEM doorways

## Abstract

**Simple Summary:**

The dissemination of cancer cells from their original location to distant organs where they grow, a process called metastasis, causes more than 90% of cancer deaths. The identification of the molecular mechanisms of metastasis and the development of anti-metastatic therapies are essential to increase patient survival. In recent years, targeting the tumor microenvironment has become a promising avenue to prevent both tumor growth and metastasis. As the tumor microenvironment contains not only cancer cells but also blood vessels, immune cells, and other non-cancerous cells, it is naïve to think that therapy only affects a single cell type in this complex environment. Here we review the importance, and ways to inhibit the function, of one therapeutic target: the receptor Tie2. Tie2 is a receptor present on the cell surface of several cell types within the tumor microenvironment and regulates tumor angiogenesis, growth, and metastasis to distant organs.

**Abstract:**

The Tie2 receptor tyrosine kinase is expressed in vascular endothelial cells, tumor-associated macrophages, and tumor cells and has been a major focus of research in therapies targeting the tumor microenvironment. The most extensively studied Tie2 ligands are Angiopoietin 1 and 2 (Ang1, Ang2). Ang1 plays a critical role in vessel maturation, endothelial cell migration, and survival. Ang2, depending on the context, may function to disrupt connections between the endothelial cells and perivascular cells, promoting vascular regression. However, in the presence of VEGF-A, Ang2 instead promotes angiogenesis. Tie2-expressing macrophages play a critical role in both tumor angiogenesis and the dissemination of tumor cells from the primary tumor to secondary sites. Therefore, Ang-Tie2 signaling functions as an angiogenic switch during tumor progression and metastasis. Here we review the recent advances and complexities of targeting Tie2 signaling in the tumor microenvironment as a possible anti-angiogenic, and anti-metastatic, therapy and describe its use in combination with chemotherapy.

## 1. Introduction

The receptor tyrosine kinases (RTKs) Tie1 (*TIE*) and Tie2 (*TEK*) were discovered in the early 1990s through a screen for tyrosine kinases expressed by endothelial cells (ECs) [1,2,3,4]. Tie is an acronym that stands for tyrosine kinase with immunoglobin and EGF homology domains. Tie1 and Tie2 share a 76% amino acid similarity in the cytoplasmic domain and a 30% amino acid identity in the extracellular domains. The cytoplasmic portion of the receptors contains a split kinase domain that binds different proteins following autophosphorylation and the extracellular portion consists of three immunoglobin (Ig)-like domains flanked by three epidermal growth factor (EGF)-like cysteine repeats and three fibronectin type III domains [5]. Although Tie2 was originally thought to be an orphan receptor, a secretion-trap cloning approach isolated angiopoietin-1 (Ang1, *ANGPT1*) as a Tie2 ligand [6]. Subsequent studies also identified Ang2 (*ANGPT2*), Ang4 (*ANGPT4*), and the mouse gene Ang3—the orthologue to human Ang4—as ligands for Tie2 [7,8,9,10,11]. Tie1 is still considered to be an orphan receptor although it can heterodimerize with Tie2 to regulate its activity [12,13]. Tie1 is expressed almost exclusively by ECs of vascular and lymphatic vessels whereas Tie2 is expressed by vascular and lymphatic ECs, endothelial progenitor cells, several cancer cells, and hematopoietic cells including macrophages. Larger vessel ECs express Tie2 more predominantly than ECs of smaller vessels and its expression is upregulated during tumor angiogenesis [2,5,14,15].

Gene targeting studies have revealed that the Ang-Tie2 system plays a critical role during both blood vessel remodeling and maturation and during the stabilization of the cardiovascular system (Table 1) [3,16,17,18]. Tie1-null mouse embryos exhibit widespread edema and hemorrhage due to decreased vessel integrity and maturation, resulting in death between E13.5 and birth [19]. Tie2-null embryos exhibit more severe phenotypes with death occurring between E9.5 and E12.5 [3,16,17]. These Tie2-deficient embryos proceed through the early stages of cardiovascular development but not beyond the capillary plexus, which is poorly organized with decreased numbers of vessel branches and a severe lack of supporting smooth muscle cells and pericytes. These embryos also display impaired hematopoiesis and heart endocardium development [3]. Embryos null for both Tie1 and Tie2 display a more severe phenotype than embryos null for Tie2 only and both receptors appear to be unnecessary for initial cardiovascular development [20]. Formation of the microvasculature during late organogenesis and postnatal hematopoiesis require both receptors [21]. Taken together, these studies indicate that although the Ang-Tie2 system is dispensable for initial vascular development, it plays an essential role in the recruitment of the supporting mural cells, vessel remodeling and maturation in the embryo, and in angiogenesis in the adult.

**Figure 1:**
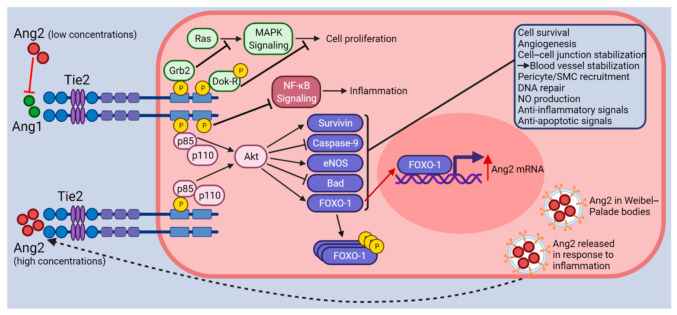
Illustration of Ang-Tie2 signaling. The Tie2 receptor undergoes autophosphorylation upon binding of Ang1. Subsequently, PI3K (p85/p110) and Akt signaling are activated, which in turn promote survival and anti-apoptotic signals through the upregulation of Survivin and eNOS and the inhibition of Caspase-9 and Bad. FOXO-1 transcription factors are involved in protein synthesis. Following Tie2 activation (effects shown with black arrows and inhibitor lines), FOXO-1 is phosphorylated and inactivated, which promotes EC quiescence, survival, and vascular stabilization. Other proteins associated with phosphorylated Tie2 such as Grb2 and Dok-R inhibit cell proliferation. ABIN-2 (not shown) associates with phosphorylated Tie2 and is thought to prevent NF-κB signaling activation through the inhibition of the IKK complex. Altogether, Tie2 activation (by Ang1 or high concentrations of Ang2) leads to vessel stabilization through EC survival, quiescence, and the stabilization of cell–cell junctions. When Ang2 binds to Tie2 at lower concentrations (effects shown with red arrows and inhibitor lines), it acts as a Tie2 antagonist preventing Ang1 from binding to Tie2. Acting as an antagonist to Tie2 signaling, Ang2 binding causes vessel destabilization, pericyte/SMC drop-off, cell migration, inflammation, and vascular leakage. Under these conditions, the FOXO-1 transcription factors are activated and promote the transcription of Ang2 mRNA, vascular destabilization, and apoptosis. Finally, the Ang2 protein produced is stored in Weibel–Palade bodies, ready for release into the ECM upon the detection of inflammatory signals. Figure created with BioRender (accessed on 15 November 2021).

Ang1 is expressed by perivascular cells such as smooth muscle cells, pericytes, fibroblasts, and tumor cells and its expression can be induced by VEGF-A, PDGF-B, and hypoxia [25,26]. Upon Ang1 binding to the Tie2 receptor, five tyrosine residues within the intracellular kinase domain of Tie2 become autophosphorylated. The activation of Tie2 stimulates several signaling pathways, most notably the PI3K pathway that stimulates EC survival, quiescence, barrier integrity, and nitric oxide synthesis through the downstream activation of protein kinase B/Akt, MAPK, eNOS, and Dok-R signaling (Figure 1) [27,28]. Ang1 and Tie2-null animals display similar phenotypes as do Ang2-overexpressing animals [7,16]. This observation led to the model that Ang2 acts an antagonist of Tie2. However, subsequent studies have demonstrated that Ang2 can function as a Tie2 agonist in a context-dependent manner when present at high concentrations [29,30]. Ang2 is almost exclusively expressed by ECs and although not expressed by quiescent ECs, its transcription is induced by VEGF-A and hypoxia during EC activation and angiogenesis [7,31,32,33]. Ang2 is considered to be only a partial agonist of Tie2 as its agonist activity for Tie2 is much lower than that of Ang1 [34]. Further, at lower concentrations, Ang2 is thought to be mainly an antagonist of Tie2 signaling through the displacement of the more active Ang1 ligand. Additional evidence for context-dependent Ang-Tie2 signaling was provided by the finding that Ang2 induces blood vessel regression in the absence of VEGF and promotes angiogenesis in the presence of VEGF [35].

Given that Tie2 activation, depending on the cell type and the environment, stimulates a variety of downstream signaling pathways and induces pro- and anti-tumor effects depending on the context, targeting Tie2 may have a multitude of effects. Moreover, Tie2 is expressed in several cell types within the tumor microenvironment, which complicates the prediction of therapeutic outcomes. Nevertheless, there have been attempts to target Tie2 signaling as a monotherapy and a combined therapy to block angiogenesis, induce an anti-tumorigenic immune response, and enhance the effect of chemotherapy. Here, we review the complexities behind targeting Tie2 in the tumor microenvironment.

## 2. Role of Ang-Tie2 in Tumor Angiogenesis

The Ang-Tie2 signaling pathway wields a critical and rate-limiting control over the early stages of tumor vascularization. Although Ang2 is only weakly expressed in quiescent ECs under physiological conditions, its expression—along with Tie2—is dramatically upregulated in hypoxia, which is a hallmark of the tumor microenvironment, making Ang2 an attractive candidate for anti-angiogenic therapies. To sustain tumor growth, the tumor must activate the angiogenic switch and co-opt the pre-existing host vasculature [36,37], leading to increased Ang2 production and secretion [38]. Acting as a Tie2 antagonist, this causes increased EC permeability and vessel regression leading to an increase in hypoxia, which in turn increases VEGF-A expression and causes a robust angiogenic response. Working in concert with VEGF signaling, the Ang-Tie2 system subsequently signals in a manner analogous to that occurring during physiological angiogenesis.

In the tumor vasculature, Ang2 is upregulated and its expression is correlated with malignancy in several cancers [39,40]. Ang2 can be detected at significant concentrations in the blood of tumor patients with esophageal squamous cell cancer [41], lung cancer [42], and hepatocellular carcinoma [43] and the expression of Ang2 correlates with tumor progression in melanoma [44]. The vasculature of glioblastoma tumors displays increased levels of Ang2 [45], particularly in the necrotic and hypoxic regions [46] and in vessels deficient in smooth muscle cell coverage [45]. Although Ang2 expression is increased in the tumor vasculature and with tumor progression, the results of Ang2 expression modulation have been shown to be context-dependent and complicated. This is not surprising, given that Ang2 has been demonstrated to be a dose-dependent agonist or antagonist of Tie2 and to exhibit differential signaling based on the presence or absence of VEGF-A.

The overexpression of Ang2 in a rat glioma model led to aberrant vessel formation and decreased smooth muscle cell coverage [47] whereas Ang2-neutralizing antibodies decreased tumor growth [48] and also prevented VEGF-induced EC migration during angiogenesis [49], underscoring the role of Ang2 during VEGF-induced angiogenesis. The induction of Ang2 in glioma, breast cancer, pancreatic neuroendocrine tumors, and lung carcinoma inhibited tumor growth and metastasis [50,51,52]. Ang2-deficient mice with Lewis lung carcinoma or melanoma exhibited a decreased initial tumor growth and a more mature vasculature with pericyte coverage compared with wildtype mice. However, the tumor growth rates were similar during the later stages [53]. In spontaneous mouse models of breast cancer, Ang2 inhibition led to decreased tumor angiogenesis and metastasis [54]. This could be due to the effects of Ang2 on Tie2-expressing macrophages (discussed below) as Ang2 inhibition decreases the upregulation of Tie2 in tumor-associated macrophages and their association with the tumor vasculature [54]. These studies highlight that the modulation of Ang2 expression (either up- or downregulation) causes altered mural cell coverage and vessel maturity resulting in delayed tumor growth.

The mechanisms by which the modulation of Ang2 expression affects tumor growth and metastasis are not yet fully understood. The studies described above suggest that the overall balance between Ang1 and Ang2 expression likely plays a crucial role in determining the effect on tumor angiogenesis and metastasis. Indeed, in many cancer types, tumor angiogenesis and a poor prognosis are associated with an increased ratio of Ang2 expression levels relative to Ang1 [55]. Recently, combination therapies of Ang2 inhibition and cytotoxic drugs with agents targeting tyrosine kinases or VEGF-A have shown enhanced anti-tumor effects compared with monotherapy [56]. DAAP (double anti-angiogenic protein), a chimeric decoy receptor that simultaneously blocks all angiopoietins and VEGF-A, inhibited tumor angiogenesis and metastasis [57]. These studies suggest that utilizing therapies to simultaneously inhibit both VEGF and Ang-Tie2 signaling is a promising strategy to block vascular permeability, tumor angiogenesis, and metastasis.

## 3. Role of Tie2^+^ Macrophages in Tumor Progression and Metastasis Formation

Hematopoietic cells of diverse lineages, including macrophages, contribute to tumor progression [58]. Monocytes are recruited from the bloodstream into tumors where they differentiate into tumor-associated macrophages. There, they contribute to tumor progression by blocking anti-tumor immunity and promoting angiogenesis, cell migration, invasion, and metastasis [59,60,61,62]. High numbers of macrophages in human primary tumor specimens correlate with increased tumor vascularization [63]. Specifically, a subset of tumor-associated macrophages that express Tie2 plays a crucial role in both tumor angiogenesis [64,65] and the dissemination of tumor cells from the primary tumor to secondary sites [66]. In this section, we will describe our current understanding of the function of Tie2-expressing macrophages in both tumor angiogenesis and dissemination.

### 3.1. Tie2 Macrophages Regulate Tumor Angiogenesis

As described in Section 2, during angiogenesis, tumor cells and associated immune cells recruit endothelial cells from the surrounding vasculature to form new blood vessels [67]. Interactions between these cell types trigger signaling pathways that result in the formation of new blood vessels and finally in tumor vascularization [68]. In particular, tumor-associated macrophages that express Tie2 receptors (previously thought to be restricted to endothelial and hematopoietic stem cells) migrate toward, and adhere to, sprouting blood vessels. These macrophages are essential for vascular anastomosis and the formation of a functional vessel system [65]. The elimination of Tie2 macrophages by a suicide gene impairs angiogenesis and reduces tumor growth [69]. Additionally, recent studies suggest that Tie2 macrophages contribute to metastatic relapse because, following chemotherapy treatments, they are recruited into tumors to promote vascular reconstruction [70]. The deletion of Tie2 macrophages impairs the metastatic relapse after chemotherapy and the mechanism by which Tie2 macrophages contribute to this process is becoming clearer.

### 3.2. Tie2 Macrophages Regulate Vascular Permeably and Intravasation

In addition to tumor angiogenesis, Tie2-expressing macrophages play an important role in tumor cell intravasation and metastasis [54,71,72]. Using multiphoton intravital imaging to study mammary carcinomas, it was found that tumor cell intravasation does not occur throughout the cancer-associated vascular endothelium. Rather, entry is localized to specific microanatomical doorways called TMEM (tumor microenvironment of metastasis) [71,73,74,75]. Following intravasation via TMEM doorways, the tumor cells enter the circulation and disseminate to secondary sites. Each TMEM doorway is composed of a three cell complex involving an endothelial cell, a perivascular Tie2^high^/VEGF^high^ macrophage, and a tumor cell expressing high levels of Mena (an actin-regulatory protein that influences cell cohesion and motility), all in direct and stable contact with each other [71,73,74,75]. Although many macrophage subtypes may be present in perivascular regions, only macrophages expressing high levels of Tie2 are capable of assembling functional TMEM structures [71,76]. The TMEM macrophage reduces the cohesion of the endothelial junctions in the immediate vicinity of TMEM through the transient secretion of VEGF-A. This causes localized and transient vascular opening, during which MenaINV^high^ migratory tumor cells intravasate and disseminate to secondary sites [71].

The critical role of Tie2^high^/VEGF^high^ macrophages in TMEM doorway function was further demonstrated by the conditional ablation of macrophages in PyMT transgenic mice and by VEGF deletion in the monocyte/macrophage lineage in FVB mice; in both cases, there was a dramatic inhibition of TMEM function and, consequently, of tumor cell intravasation and metastasis formation [71,77]. Additionally, these proangiogenic perivascular Tie2 macrophages can be inhibited by the Tie2 inhibitor rebastinib [78]. This offers new treatment options by targeting TMEM-associated vascular opening and tumor cell dissemination. The pharmacological inhibition of Tie2 macrophages is discussed in the following section.

TMEM doorways have been detected in human mammary carcinomas and their density is a clinically validated prognostic marker for distant recurrence in breast cancer patients [73,74,79]. Recently, it was found that TMEM doorways are also present at secondary sites in the mouse and human lung metastases of breast cancer patients [80] as well as in lymph node tumor cell nests [81]. This finding suggests that TMEM doorway-mediated tumor cell dissemination occurs not only from the primary tumor but also from the secondary sites, leading to an increased metastatic burden and hastening the death of stage IV breast cancer patients. Indeed, using photo-convertible fluorescent protein-based fate mapping, it has been possible to mark tumor cells within established lung metastases and track redisseminated tumor cells to tertiary sites such as the bone marrow [82].

### 3.3. Targeting Tie2 to Prevent Tumor Progression and Metastasis Formation

The identification of Tie2 macrophages as a key player in tumor angiogenesis, intravasation, and immunosuppression offers the possibility of combination therapies that, in addition to targeting tumor cells, target the cells of the tumor microenvironment. Although anti-angiogenic treatments such as bevacizumab and other VEGF-A pathway inhibitors have shown an initial efficacy in decreasing tumor angiogenesis and disease burden, patients rapidly develop a resistance to these agents [83,84]. This is due to tumor infiltration by immune cells, especially Tie2-expressing macrophages, in response to hypoxia and cell death after vascular regression [84]. Adding agents that target Ang-Tie2 signaling between ECs and macrophages such as Tie2-neutralizing antibodies, siRNA-targeting Tie2, and inducible Tie2 knockdown in the hematopoietic stem cells in addition to anti-angiogenic agents has yielded promising pre-clinical results, blocking tumor angiogenesis, growth, and metastasis [54].

As tumor cells disseminate via the TMEM doorways [66,75], the pharmacological inhibition of the TMEM doorways to seal them to tumor cell intravasation is a very promising novel therapeutic approach to block intravasation, the dissemination of tumor cells, and, ultimately, metastasis formation. As previously described, the TMEM doorway is composed of a perivascular Tie2^high^/VEGF^high^ macrophage, a Mena-expressing tumor cell, and an endothelial cell, all in direct stable contact with each other [71,73,74,75]. To date, there is not a specific pharmacological approach to target Mena-expressing tumor cells nor a specific approach to block the association of ECs with TMEM doorways. Therefore, blocking the Tie2 macrophage is the most promising strategy to seal the TMEM doorways to intravasation.

We have described rebastinib, a novel and highly specific switch pocket inhibitor of Tie2 tyrosine kinase with picomolar potency [78]. Recent pre-clinical studies of metastatic mammary carcinoma and pancreatic neuroendocrine tumors in orthotopic mouse models showed that rebastinib reduces tumor growth, angiogenesis, and metastasis and significantly increases the overall survival of paclitaxel-treated mice even after the resection of the primary tumor [78]. By inhibiting Tie2 in tumor-associated macrophages, this drug prevented the VEGF-dependent vascular opening associated with the TMEM doorways. Consequently, rebastinib reduced the circulating tumor cells and their dissemination to the lungs [78,85]. Rebastinib is now being tested in several clinical trials; three in solid tumors and one in leukemias. NCT02824575 is a phase 1b trial for breast cancer in women with HER2-negative adenocarcinoma of the breast, metastatic breast cancer, or women with prior chemotherapy and/or endocrine therapy where rebastinib is being used in combination with an anti-tubulin therapy, paclitaxel, or eribulin. NCT03717415 is phase 1b/2 trial for patients with locally advanced or metastatic solid tumors testing rebastinib in combination with carboplatin. The inclusion criterion for the dose escalation phase (Part 1) is patients who have been diagnosed with locally advanced or metastatic solid tumors for which carboplatin, an alkylating agent, is considered to be an appropriate treatment. The dose expansion phase (Part 2) includes patients with previously treated triple negative breast cancer, recurrent platinum-sensitive ovarian cancer, or pleural or peritoneal malignant mesothelioma. NCT03601897 is a phase 1b/2 trial for patients with locally advanced or metastatic solid tumors for which paclitaxel is considered to be an appropriate treatment. Part 2 of the trial will be completed next year and includes patients with triple negative and stage IV inflammatory breast cancer, recurrent ovarian cancer, recurrent or metastatic endometrial cancer, or advanced gynecological carcinosarcoma.

Several other small-molecule tyrosine kinase inhibitors that target Tie2 along with VEGFR2 and other RTKs have been tested in recent clinical trials. Ripretinib is a c-Kit, VEGFR2, PDGFRα, and Tie2 inhibitor approved for use as a salvage therapy for patients with advanced gastrointestinal stroma tumors who have received prior treatments with three or more kinase inhibitors (INVICTUS/NCT03353753) [86,87]. Regorafenib is a multikinase inhibitor of Tie2, VEGFR1/2/3, and FGFRs and has achieved approval as a salvage therapy for metastatic colorectal cancer, advanced gastrointestinal stromal tumors, and advanced hepatocellular carcinoma (NCT01103323) [88].

The identification of functional TMEM doorways not only in primary tumors but also in secondary metastatic sites demonstrates that TMEM doorways are not restricted solely to the primary tumor microenvironment but are additionally implicated in tumor cell redissemination from metastatic sites. These studies suggest that TMEM doorways are a common mechanism of tumor cell dissemination during all stages of breast cancer progression, indicating that it is never too late to block dissemination and to treat metastatic patients [80,81,82].

In conclusion, these studies underscore that targeting Tie2 macrophages in combination with therapies targeting tumor cells is a novel and promising approach to block tumor progression and decrease the metastatic burden. Additionally, a major advantage in targeting macrophages is that macrophages are genetically stable cells and, unlike tumor cells, are less likely to develop a drug resistance.

## 4. Role of Tie2 Expression in Cancer Cells

Only a small number of studies have reported Tie2 expression in tumor cells of both an epithelial and a non-epithelial origin. Kukk et al., (1997) investigated Tie2 signaling in hematopoietic progenitors and leukemic cells. The group reported Tie2 expression in a subset of leukemic blasts despite the fact that the peripheral mononuclear cells did not express Tie2 [89]. This review will focus on the role of Tie2 in non-hematologic malignancies.

Along these lines, Mitsutake et al., (2002) demonstrated the expression of Tie2 in thyroid tumor cells using a combination of gene expression analysis (RT-PCR), immunohistochemistry (IHC), in situ hybridization (ISH), and laser capture microdissection (LCM) in human tumors and thyroid cancer cell lines [90]. However, no notable studies on the importance of Tie2 signaling in thyroid cancer have since been published. In another study, Chen et al., (2016) suggested that paracrine factors secreted from cancer-associated fibroblasts (CAFs) promote the Ang1/Tie2 signaling axis in breast tumor cells, eventually leading to increased survival and proliferation in vitro [91]. However, no direct in vivo evidence was given with regard to Tie2 signal propagation in breast cancer cells. The exact role of Tie2^+^ tumor cells in cancer development and progression remains to be elucidated.

High Tie2 expression has also been shown to be a prognostic factor for poor survival and increased metastases in several cancers. In high grade serous ovarian cancer, high Tie2 expression predicted a shorter overall survival and distal omental metastasis. Tie2 expression was also increased in metastatic lesions compared with primary tumors [92]. In gastric cancer, an evaluation of Tie2 expression through immunohistochemistry found that Tie2 expression increased with increasing tumor grade, the presence of lymph node metastases, higher recurrence rates, and poor patient survival [93]. Tumor cell-derived Ang2 has also recently been implicated as a promoter of metastatic colonization in melanoma. Abdul Pari et al., (2020) showed a subset of melanoma patients had strong expression of Ang2 in tumor cells in the primary tumor and this was correlated with the metastatic progression [94]. The expression of Ang2 was inversely correlated with survival in patients with melanoma. By silencing the expression of Ang2 in the tumor cells, the authors found that Ang2 expression was dispensable for primary tumor growth but was important for the metastatic colonization of tumor cells. Global transcriptional profiling of the Ang2-deficient tumor cells uncovered a defect in the ability of the tumor cells to respond to oxidative stress and increased MAPK signaling, indicating that increased expression of Ang2 in tumor cells can enhance metastatic colonization by protecting the tumor cells from stressors within the tumor microenvironment [94].

In the nervous system, Lee et al., (2006) demonstrated a subpopulation of neoplastic Tie2^+^ glial cells that could adhere to collagens type I and IV in an Ang1/Tie2-dependent manner, facilitating the process of glial cell invasion and migration [95]. More substantial evidence that Tie2 signaling may be involved in glioma cell invasion has been shown in a recent study by Tilak et al., (2021) in which the interaction between the receptor Tie2 and the adaptor protein ShcD/ShC4 synergistically promoted invadopodium formation, matrix degradation, and subsequently glioma cell invasion [96]. It was also demonstrated that the co-expression of Tie2 and ShcD/SHC4 in invasive glioma cells correlated with an increased expression of N-Cadherin [96], a classical marker of epithelial-to-mesenchymal transition (EMT) in cancer that supports mesenchymal and invasive phenotypes [97]. Although Tie2 ligands Ang1 and Ang2 both seem to play important roles in Tie2 activation and cancer progression in gliomas, Brunckhorst et al., (2010) previously showed that Ang4 is an equally vital factor promoting glioblastoma progression in a Tie2-dependent manner [98]. However, it is not clear whether Ang4 promotes glioblastoma progression directly (by affecting Tie2 signaling in glioblastoma cells) or indirectly (via enhanced angiogenesis and survival signaling in the tumor microenvironment) [98].

Signaling through the Tie2 receptor in nervous system tumors, particularly glioblastomas, has also been associated with the induction of DNA repair pathways especially after genotoxic therapies. Following sublethal ionizing radiation for example, Hossain et al., (2016), showed that Tie2 may translocate into the nucleus whereby it promotes a radioresistant phenotype through a non-homologous end-joining mechanism of DNA repair [99]. Mechanistically, this is mediated via the Tie2-dependent phosphorylation of histone H4 and the consequent recruitment of the proto-oncogene ABL1 to the DNA repair complex [99]. In our view, this surprising role of Tie2 in promoting radioresistance in glioblastoma cells is accompanied by an even more paradoxical localization and function of Tie2 within the nucleus of tumor cells. In an effort to describe this phenomenon in more detail, Hossain et al., (2017) explained that irradiation-induced Tie2 trafficking is dependent on the endocytic pathway involving caveolin-1, the principal component of caveolae [100]. Indeed, the specific targeting of caveolin-1, either via genetic ablation or pharmacologic inhibition, promoted radiosensitivity in glioblastoma cells [100]. Interestingly, Tie2-mediated radioresistance was originally reported in Tie2^+^ ECs in the bone marrow niche whereby they worked with hematopoietic stems cells to repopulate the repertoire of bone marrow-resident hematopoietic progenitors following ionizing radiation [101]. Taken together, these observations suggest that certain tumor cell types can hijack the Tie2-dependent response machinery as a means of promoting survival and achieving the critical hallmarks of cancer development and progression.

## 5. Conclusions

Ang-Tie2 signaling is vital for the physiological regulation of endothelial cell permeability, vascular maturity, and angiogenesis and also for many cell–cell interactions within the tumor microenvironment. Here, we summarized the current knowledge of Tie2 signaling in several of the major cells that make up the tumor milieu and described the promising approach of targeting this signaling pathway to thwart the metastatic cascade. The Ang-Tie2 system provides an appropriate target for several critical cell types and their intercommunication within the tumor microenvironment, causing anti-angiogenic and anti-metastatic effects. Moreover, targeting the Ang-Tie2 signaling system may be particularly useful to enhance the effects of chemotherapeutics on cancer progression. The use of anti-Tie2 therapies has been so far restricted to the treatment of patients with late-stage disease or after the failure of several other therapies to varying effects. Future studies should investigate the use of anti-Tie2 treatments as a combined therapy for the treatment of early-stage diseases. Given the highly context-dependent effects of manipulating Ang-Tie2 signaling, it is crucial that future studies investigate Ang-Tie2 signaling between the ECs, immune cells, and tumor cells altogether as well as in the context of VEGF signaling. The uncovering of the molecular mechanisms governing Ang-Tie2 signaling in the tumor microenvironment is a critical milestone in designing therapies to decrease the systemic cancer burden and increase the survival of patients with metastatic disease.

## Figures and Tables

**Table 1:** Phenotypes of Ang-Tie2 knockout mice.

Genotype	Phenotype	References
Tie1*^−/−^*	Lethality between E13.5 and birth; loss of vascular integrity and maturity; widespread edema and hemorrhaging	[19]
Tie2^−/−^	Embryonic lethality between E9.5 and E12.5; vessel remodeling defects in the brain and plexus of the yolk sac; severe heart defects; decreased numbers of vascular ECs; poorly organized vessels with fewer branches and less pericyte coverage	[3,16,17,18,22]
Tie1^−/−^; Tie2^−/−^	Embryonic lethality at E10.5; vasculature develops normally; cardiovascular defects; loss of vascular integrity and maturity	[21]
Ang1^−/−^	Embryonic lethality between E11 and E12.5; growth-retarded hearts; collapsed endocardium and endothelial lining in the atria; immature primary plexus; decreased pericyte coverage of vessels	[16]
Ang2^−/−^	Lethality by postnatal day P14; chylous ascites develop a few days after birth; severe vascular defects including collapsed endocardial lining from the myocardium and disruption of vascular integrity; impaired response to inflammatory challenges	[23,24]
